# Bacteremia is an independent risk factor for mortality in nosocomial pneumonia: a prospective and observational multicenter study

**DOI:** 10.1186/cc10036

**Published:** 2011-02-16

**Authors:** Mònica Magret, Thiago Lisboa, Ignacio Martin-Loeches, Rafael Máñez, Marc Nauwynck, Hermann Wrigge, Silvano Cardellino, Emili Díaz, Despina Koulenti, Jordi Rello

**Affiliations:** 1 Critical Care Department, Sant Joan University Hospital, Rovira i Virgili University, Pere Virgili Health Institut, Sant Joan St, Reus 43201, Spain; 2 Critical Care Department, Joan XXIII University Hospital, Rovira i Virgili University, Pere Virgili Health Institut and CIBER Enfermedades Respiratorias (CIBERES), Mallafré Guasch St, Tarragona 43005, Spain; 3 Critical Care Department, Master Misericordiae University Hospital, Eccles Street, Dublin 7, Ireland; 4 Critical Care Department, Bellvitge University Hospital, Calle Feixa Llarga, Hospitalet de Llobregat 08907, Spain; 5 Critical Care Department, St Jan Hospital, Ruddershove Street, Brugge 8000, Belgium; 6 Department of Anesthesiology and Intensive Care Medicine, University Hospital Bonn, Wilhelmstraße, Bonn 53111, Germany; 7 Critical Care Department, Cardinal Massaia Hospital, Ospedali Riuniti Strada, Asti 14100, Italy; 8 Critical Care Department, University General Hospital Attikon, Rimini, Haidari 12462, Greece; 9 Critical Care Department, Vall d'Hebron University Hospital, CIBERES, VHIR, Universitat Autonoma de Barcelona, Vall d'Hebron St, Barcelona 08035, Spain

## Abstract

**Introduction:**

Since positive blood cultures are uncommon in patients with nosocomial pneumonia (NP), the responsible pathogens are usually isolated from respiratory samples. Studies on bacteremia associated with hospital-acquired pneumonia (HAP) have reported fatality rates of up to 50%. The purpose of the study is to compare risk factors, pathogens and outcomes between bacteremic nosocomial pneumonia (B-NP) and nonbacteremic nosocomial pneumonia (NB-NP) episodes.

**Methods:**

This is a prospective, observational and multicenter study (27 intensive care units in nine European countries). Consecutive patients requiring invasive mechanical ventilation for an admission diagnosis of pneumonia or on mechanical ventilation for > 48 hours irrespective of admission diagnosis were recruited.

**Results:**

A total of 2,436 patients were evaluated; 689 intubated patients presented with NP, 224 of them developed HAP and 465 developed ventilation-acquired pneumonia. Blood samples were extracted in 479 (69.5%) patients, 70 (14.6%) being positive. B-NP patients had higher Simplified Acute Physiology Score (SAPS) II score (51.5 ± 19.8 vs. 46.6 ± 17.5, *P *= 0.03) and were more frequently medical patients (77.1% vs. 60.4%, *P *= 0.01). Mortality in the intensive care unit was higher in B-NP patients compared with NB-NP patients (57.1% vs. 33%, *P *< 0.001). B-NP patients had a more prolonged mean intensive care unit length of stay after pneumonia onset than NB-NP patients (28.5 ± 30.6 vs. 20.5 ± 17.1 days, *P *= 0.03). Logistic regression analysis confirmed that medical patients (odds ratio (OR) = 5.72, 95% confidence interval (CI) = 1.93 to 16.99, *P *= 0.002), methicillin-resistant *Staphylococcus aureus *(MRSA) etiology (OR = 3.42, 95% CI = 1.57 to 5.81, *P *= 0.01), *Acinetobacter baumannii *etiology (OR = 4.78, 95% CI = 2.46 to 9.29, *P *< 0.001) and days of mechanical ventilation (OR = 1.02, 95% CI = 1.01 to 1.03, *P *< 0.001) were independently associated with B-NP episodes. Bacteremia (OR = 2.01, 95% CI = 1.22 to 3.55, *P *= 0.008), diagnostic category (medical patients (OR = 3.71, 95% CI = 2.01 to 6.95, *P *= 0.02) and surgical patients (OR = 2.32, 95% CI = 1.10 to 4.97, *P *= 0.03)) and higher SAPS II score (OR = 1.02, 95% CI = 1.01 to 1.03, *P *= 0.008) were independent risk factors for mortality.

**Conclusions:**

B-NP episodes are more frequent in patients with medical admission, MRSA and *A. baumannii *etiology and prolonged mechanical ventilation, and are independently associated with higher mortality rates.

## Introduction

Since positive blood cultures are uncommon in nosocomial pneumonia (NP) patients, the responsible pathogens are usually isolated from respiratory samples [1-3]. Studies on bacteremia associated with hospital-acquired pneumonia (HAP) have reported fatality rates up to 50% [4,5]. Although the impact of methicillin resistance on the outcomes of patients with *Staphylococcus aureus *bacteremia has been extensively evaluated, little information exists on the impact of the methicillin resistance of patients with nosocomial bacteremic *S. aureus *pneumonia. A prospective study in a single institution reported recently that methicillin-resistant *S. aureus *(MRSA) was associated with bacteremic ventilator-associated pneumonia (VAP) and that bacteremia significantly increased mortality in these patients [6]. Whether these findings are generalizable to other case mixes or institutions is unknown.

The response to VAP can be shown from compartmentalized forms that account for a local response with minor systemic compromise, whereas systemic spillover or escape of inflammation led to septic shock and bacteremia. Moreover, some microorganisms such as *S. aureus *are more adherent than others [7] and are more likely to develop bacteremia.

Because some intensive care units (ICUs) do not perform blood cultures as part of the diagnosis work in patients with suspected NP and this information provides useful epidemiologic information on causative organisms and resistance, we performed a secondary analysis of a large multicenter cohort of patients with NP [8]. The primary objective was to confirm whether bacteremic nosocomial pneumonia (B-NP) had higher mortality rates than nonbacteremic nosocomial pneumonia (NB-NP). Secondary objectives were to identify which risk factors and pathogens were associated with development of B-NP.

## Materials and methods

### Study population and design

The EU-VAP/CAP was a prospective, observational survey conducted in 27 ICUs from nine European countries (Belgium, France, Germany, Greece, Italy, Ireland, Portugal, Spain and Turkey). The principal investigator contacted one coordinator in each country (national coordinator) who then selected the participating centers for its country. All patients requiring admission for a diagnosis of pneumonia or on invasive mechanical ventilation for longer than 48 hours, irrespective of the diagnosis at admission, were included.

The target was the collection of data for 100 consecutive admissions in each ICU. Data were collected by the primary investigator in each site (see Acknowledgements for list of investigators). The period of data collection was between 6 and 12 months (depending on the size and type of the participating ICUs). Patient demographics, primary diagnosis, ICU and hospital lengths of stay, Simplified Acute Physiology Score (SAPS) II score [9], duration of mechanical ventilation and outcome (ICU mortality) were recorded for all patients.

Each clinical episode of pneumonia was described separately. For patients with a clinical diagnosis of pneumonia, data collection included clinical signs, sepsis severity (sepsis/severe sepsis/septic shock) [10] and Sepsis-related Organ Failure Assessment score [11] for the day of admission to the ICU for community-acquired pneumonia and HAP, and for the day of clinical suspicion for VAP and microbiology.

The present study was approved by the Ethics Board of the coordinating center (Clinical Research Ethics Committee, Joan XXIII University Hospital, Tarragona, Spain). The participating centers either received ethical approval from their institutions or ethical approval was waived. Informed consent was waived due to the observational nature of the study.

### Definitions

Pneumonia was diagnosed when new, persistent pulmonary infiltrates, not otherwise explained, appeared on chest radiographs with the presence of local (purulent respiratory secretions) and systemic signs of inflammatory response (white blood cell count > 10,000/μl, or increase in white blood cell count > 20% in the absence of leukocytosis or fever).

Bacteremic pneumonia was defined as at least one positive blood culture not related to another source of infection and at least one positive respiratory sample culture (obtained within 48 hours of each other if two or more cultures). In addition, at least one of the microorganisms isolated in respiratory samples had to be isolated in blood cultures, whereas all isolates in blood cultures were required to grow in simultaneously obtained respiratory samples to fit the complete definition of bacteremic pneumonia. This was only diagnosed when respiratory and blood samples yielded the same microorganism and both cultures were performed within 48 hours. Other growths in both respiratory and blood cultures within this period were defined as inconsistent microbiology.

Fever was defined as two or more consecutive measurements > 38°C. Pneumonia was considered ventilator-associated when it occurred 48 hours after starting mechanical ventilation, and was defined as early-onset if it started within 4 days of admission, in accordance with the American Thoracic Society/Infectious Disease Society of America guidelines [12]. Trauma was defined as the presence of injury in more than one body area or system, or the presence of major cranial trauma alone. Prior antibiotic exposure was considered when a patient received antimicrobial agents during the 15 days preceding the NP episode, with the exception of antibiotics administered for surgical prophylaxis [13]. Shock was described as systolic blood pressure < 90 mmHg despite adequate fluid resuscitation and need for vasopressor agents. At least 48 hours of hospitalization in the 90 days before admission or current hospitalization for > 4 days before the start of mechanical ventilation was considered as prior hospitalization.

### Microbiology

Quantitative or qualitative tracheal aspirates or bronchoscopic examination using bronchoscopic-protected specimen brush samples or bronchoscopic bronchoalveolar lavage samples was performed to obtain uncontaminated lower airway secretions for bacterial cultures. Bacterial identification and susceptibility testing were performed by standard methods.

### Statistical analysis

Discrete variables were expressed as counts (percentage) and continuous variables as the mean and standard deviation, unless stated otherwise; all statistical tests were two-sided. The threshold for statistical significance was defined as *P *< 0.05. Differences in categorical variables were calculated using a two-sided likelihood ratio chi-square test or Fisher exact test, and the Mann-Whitney U test or Kruskal-Wallis test were used for continuous variables, when appropriate.

Backward logistic regression was used to assess the risk factors for bacteremia. Variables significantly associated with mortality in the univariate analysis were entered into the model. In order to avoid spurious associations, variables entered into the regression models were those with a relationship in univariate analysis (*P *≤ 0.05) or a plausible relationship with the dependent variable. Potential explanatory variables were checked for collinearity prior to inclusion in the regression models using tolerance and the variance inflation factor. Variables associated with bacteremia in univariate analysis were included in a multivariate analysis for identification of independent variables after adjustment for severity of disease using SAPS II. To assess the effect of bacteremia on mortality, a stepwise logistic regression was performed adjusting for admission category and severity of illness (SAPS II). Results are presented as the odds ratio (OR) and 95% confidence interval (CI). Data analysis was performed using SPSS for Windows 13.0.0 (SPSS, Chicago, IL, USA).

## Results

A total of 2,436 intubated patients were evaluated, and 689 developed NP (465 VAP and 224 HAP). Blood samples were extracted in 479 (69.5%) patients, and 70 (14.6%) of them were positive. Clinical and epidemiological data for the current study cohort are detailed in Table 1. No significant differences were observed between B-NP patients and NB-NP patients regarding age and male gender (59.2 ± 15.4 years vs. 56.5 ± 18.9 years, *P *= 0.26 and 71.4% vs. 68%, *P *= 0.67, respectively), but B-NP patients had higher SAPS II score than NB-NP patients (51.5 ± 19.8 vs. 46.6 ± 17.5, *P *= 0.03). In terms of diagnostic category, B-NP patients were more frequently medical patients than NB-NP patients (77.1% vs. 60.4%, *P *= 0.01). B-NP patients had more elapsed time between ICU admission and VAP than NB-NP patients (7.3 ± 14.1 days vs. 4.9 ± 5.8 days, *P *= 0.02).

**Table 1:** Clinical and epidemiological characteristics of bacteremic and nonbacteremic nosocomial pneumonia patients

Characteristic	Bacteremic (*n *= 70)	Nonbacteremic (*n *= 409)	*P *value
Age (years)	59.2 ± 15.4	56.5 ± 18.9	0.26
Male gender	50 (71.4)	278 (68)	0.67
SAPS II score	51.5 ± 19.8	46.6 ± 17.5	0.03
Gap pneumonia	7.3 ± 14.1	4.9 ± 5.8	0.02
Diagnostic category at admission			0.01
Medical	54 (77.1)	246 (60.4)	
Surgery	12 (17.1)	64 (15.7)	
Trauma	4 (5.7)	97 (23.8)	
Co-morbidities at admission			
Diabetes mellitus	3 (4.3)	16 (3.9)	0.75
Hepatic cirrhosis	3 (4.3)	10 (2.4)	0.42
COPD	5 (7.1)	21 (5.1)	0.57
Chronic renal failure	10 (14.3)	32 (7.1)	0.34
CCI	9 (12.9)	29 (7.1)	0.15
Alcohol	0 (0)	17 (4.2)	0.15
Immunodepression	6 (8.6)	16 (3.9)	0.11
Relating to episode at admission			
Septic shock	27 (39.7)	137 (35)	0.35
Prior antibiotic exposure	15 (21.2)	53 (13)	0.07

Data presented as mean ± standard deviation or *n *(%). SAPS, Simplified acute physiology score; Gap pneumonia, time between intensive care unit admission and ventilator-associated pneumonia; COPD, chronic obstructive pulmonary disease; CCI, congestive cardiac insufficiency.

No significant differences were observed in co-morbidities between B-NP patients and NB-NP patients. Although there were no differences in baseline co-morbidities between B-NP patients and NB-NP patients and the SAPS II score on the day of ICU admission was higher in surgical patients than medical and trauma patients (51.1 ± 17.3 vs. 48.4 ± 18.3 vs. 41.1 ± 15.7, *P *< 0.001), in the period prior to developing NP medical patients had a higher SAPS II score than surgical and trauma patients (44.9 ± 17.1 vs. 41.8 ± 15 vs. 38.5 ± 17.6, *P *< 0.02). A nonsignificant trend to positive blood cultures was associated with prior antibiotic exposure (21.2% vs. 13%, *P *= 0.07). No differences were found regarding septic shock (39.7% vs. 35%, *P *= 0.35). No difference was found in performance of the diagnostic technique between B-NP and NB-NP.

ICU mortality was significantly higher in B-NP patients compared with NB-NP patients (57.1% vs. 33%, *P *< 0.001). B-NP patients had a more prolonged mean ICU length of stay after pneumonia onset than NB-NP patients (28.5 ± 30.6 days vs. 20.5 ± 17.1 days, *P *= 0.03) (Table 2).

**Table 2:** Outcomes in bacteremic and nonbacteremic nosocomial pneumonia patients

	Bacteremic	Nonbacteremic	*P *value
ICU mortality	40 (57.1)	135 (33)	< 0.001
Survivors' length of ICU stay after pneumonia onset (days)	28.5 ± 30.6	20.5 ± 17.1	0.03
Survivors' days of MV after pneumonia onset (days)	19.5 ± 30.9	14 ± 15.7	0.11

Data presented as mean ± standard deviation or *n *(%). ICU, intensive care unit; MV, mechanical ventilation.

The pathogens isolated in blood cultures of B-NP are presented in Table 3. The main pathogen isolated in blood cultures of B-NP patients was MRSA (22.6%) followed by *Acinetobacter baumannii *(17.9%). Respiratory isolates for B-NP and NB-NP are detailed in Table 4. The most prevalent pathogen in B-NP patients was *A. baumannii *followed by MRSA. In contrast, the most prevalent pathogen in NB-NP patients was *Pseudomonas aeruginosa *followed by methicillin-susceptible *S. aureus*.

**Table 3:** Organisms isolated in blood cultures of patients with bacteremic nosocomial pneumonia

Isolate	*n *(%)
Gram-positive	
Methicillin-resistant *Staphylococcus aureus*	19 (22.6)
Methicillin-susceptible *Staphylococcus aureus*	11 (13.1)
*Streptococcus pneumoniae*	2 (2.4)
Gram-negative	
*Acinetobacter baumannii*	15 (17.9)
*Pseudomonas aeruginosa*	12 (14.3)
*Klebsiella *species	11 (13.1)
*Escherichia coli*	7 (8.3)
*Enterobacter *species	5 (5.9)
*Proteus mirabilis*	1 (1.2)
*Serratia *species	1 (1.2)

**Table 4:** Isolates in respiratory samples of bacteremic and nonbacteremic nosocomial pneumonia episodes

Isolate	Bacteremic (*n *= 117)	Nonbacteremic (*n *= 378)	*P *value
Gram-positive			
MSSA	13 (11.1)	55 (15.6)	0.29
MRSA	21 (18)	48 (12.7)	0.19
*Streptococcus pneumoniae*	2 (1.8)	17 (4.5)	0.29
Gram-negative			
*Haemophilus influenzae*	2 (1.8)	22 (5.8)	0.13
*Pseudomonas aeruginosa*	17 (14.5)	67 (17.7)	0.51
*Acinetobacter baumannii*	22 (18.8)	47 (12.4)	0.11
*Escherichia coli*	8 (6.8)	39 (10.3)	0.34
*Enterobacter *species	7 (6)	22 (5.8)	0.89
*Klebsiella pneumoniae*	15 (12.8)	22 (5.8)	0.02
*Proteus *species	2 (1.8)	9 (2.4)	0.98
*Serratia *species	1 (1)	7 (1.9)	0.8
*Moraxella *species	0 (0)	1 (0.3)	0.12
*Stenotrophomonas maltophilia*	4 (3.5)	9 (2.4)	0.75
*Morganella morgagnii*	0 (0)	2 (0.5)	0.03
*Citrobacter *species	0 (0)	3 (0.8)	< 0.99
*Burkholderia cepacia*	0 (0)	1 (0.3)	0.12
Other GNB	1 (1)	5 (1.3)	< 0.99
Other anaerobic	1 (1)	2 (0.5)	0.09

Data presented as *n *(%). MRSA, methicillin-resistant *Staphylococcus aureus*; MSSA, methicillin-susceptible *Staphylococcus aureus*; GNB, Gram negative bacteria.

To identify independent risk factors for bacteremia, a backward logistic regression included diagnostic category, MRSA and *A. baumannii *etiology, duration of mechanical ventilation, SAPS II score and prior antibiotic use. The model showed (Table 5) that medical patients (OR = 5.72, 95% CI = 1.93 to 16.99, *P *= 0.002) and surgical patients (OR = 5.06, 95% CI = 1.47 to 17.47, *P *= 0.01), compared with trauma patients, MRSA (OR = 3.42, 95% CI = 1.57 to 5.81, *P *= 0.01), *A. baumannii *(OR = 4.78, 95% CI = 2.46 to 9.29, *P *< 0.001) and duration of mechanical ventilation (OR = 1.02 per day, 95% CI = 1.01 to 1.03, *P *< 0.001), were independently associated with B-NP episodes.

**Table 5:** Binomial logistic regression (multivariate) analysis of risk factors associated with bacteremic nosocomial pneumonia

Variable	Wald value	Exp(*B*) (95% confidence interval)	*P *value
Constant	49.389		
Diagnostic category			
Medical	9.86	5.72 (1.93 to 16.99)	0.002
Surgical	6.58	5.06 (1.47 to 17.47)	0.01
Trauma	1		
SAPS II	2.995	1.01 (0.99 to 1.03)	0.08
MRSA etiology	10.958	3.42 (1.57 to 5.81)	0.01
*Acinetobacter *etiology	21.287	4.78 (2.46 to 9.29)	< 0.001
Duration of MV	12.434	1.02 (1.01 to 1.03)	< 0.001

SAPS II, Simplified Acute Physiology Score; MRSA, methicillin-resistant *Staphylococcus aureus*; MV, mechanical ventilation.

A backward logistic regression to identify independent risk factors for mortality showed that bacteremia was an independent risk factor for ICU mortality (OR for death = 2.01, 95% CI = 1.22 to 3.55, *P *= 0.008) after adjustment for severity of illness. The SAPS II score (OR for death = 1.02 per point, 95% CI = 1.01 to 1.03, *P *= 0.008) and diagnostic category (medical patients (OR for death = 3.71, 95% CI = 2.01 to 6.95, *P *= 0.02) and surgical patients (OR for death = 2.32, 95% CI = 1.10 to 4.97, *P *= 0.03)) were also independent variables associated with ICU mortality (Table 6).

**Table 6:** Binomial logistic regression (multivariate) analysis of risk factors associated with mortality in bacteremic nosocomial pneumonia

Variable	Wald value	Exp(*B*) (95% confidence interval)	*P *value
Constant	39.707		
Diagnostic category			
Medical	3.65	3.71 (2.01 to 6.95)	0.02
Surgical	2.34	2.32 (1.10 to 4.97)	0.03
Trauma	1		
SAPS II	7.033	1.02 (1.01 to 1.03)	0.008
Gap pneumonia	0.518	1.01 (0.98 to 1.04)	0.472

SAPS II, Simplified Acute Physiology Score; Gap pneumonia: time between intensive care unit admission and ventilator-associated pneumonia.

## Discussion

The present analysis of a large, cohort, prospective, multicenter research study of NP reports that bacteremic episodes cause ICU mortality to be twice that of NB-NP patients. MRSA and *A. baumannii *(and medical condition on admission compared with trauma) were identified as independent risk factors for developing bacteremia.

To our knowledge, this is the first prospective study examining bacteremic episodes in critically ill patients requiring mechanical ventilation due to NP. The present study reports that 14.6% of NP episodes in European ICUs have bacteremia. Our prevalence is within the range (8 to 20%) of previous studies that included all patients with NP not admitted to the ICU [14,15], but is lower than that (17.3%) shown in the study of Agbaht and colleagues that only included ICU patients with VAP diagnosis [6].

The response to VAP might vary from compartmentalized forms that account for a local response with minor systemic compromise, whereas systemic spillover or escape of inflammation led to septic shock and bacteremia. VAP is characterized by an exuberant increase in procoagulant activity, which precedes the clinical diagnosis of VAP [16]. A well-known fact, confirmed by *in vitro *studies, is that *S. aureus has *a propensity to cause bacteremia. These studies have demonstrated that *S. aureus *is more adherent than other microorganisms because it exhibits a high adherence manifested by the interaction of plasma fibrinogen with the fibrinogen-binding proteins (the clumping factor) [7]. Strains carrying the clumping factor are known to cause more invasive diseases [17]. As fibrinogen is an acute-phase reactant that is frequently elevated in critically ill patients, increased levels of this molecule have been proposed to potentially increase its adsorption onto the endothelial surface in susceptible patients, thereby allowing more *S. aureus *to adhere through the fibrinogen receptor [18]. Moreover, fibrin deposits enhance inflammatory responses by increasing vascular permeability, activating endothelial cells to produce proinflammatory mediators, and eliciting recruitment and activation of neutrophils. One alternative explanation may include the immunomodulating properties of *S. aureus*. This pathogen constitutively has the possibility to release enterotoxins that show superantigen activity and effectively modify the functions of various inflammatory cells [19,20]. This stimulation may lead to inflammation, aggravating airway disease in both the upper and lower respiratory tracts. In our study, higher SAPS II score and bacteremia were associated with high mortality rates that could be explained by an abnormal inflammatory response which was associated with poor outcomes.

The main pathogen isolated in blood samples of B-NP patients was *S. aureus *(35.7%), including methicillin-susceptible *S. aureus *and MRSA, followed by *A. baumannii *(17.9%). These results represent the same distribution that Agbaht and colleagues reported in a matched case-control study comparing bacteremic VAP versus nonbacteremic VAP episodes [6]. *S. aureus *was the pathogen most commonly associated with bacteremia. This pathogen was also the most prevalent microbial etiology (27%) in a prospective study of B-NP [13] and the most prevalent (24%) in a cohort of 112 ICU patients with B-NP [21].

The microbial etiology of HAP affected bacteremia development, since both *A. baumannii *and, to a lesser extent, MRSA were identified as independent predictors of bacteremia even after adjustment for confounders. *A. baumannii *exhibits an intrinsic resistance to multiple antimicrobial agents and generates a continuing controversy about whether VAP caused by this microorganism increases morbidity and mortality independently of the effect of other confounding factors in the ICU setting [22-25]. In contrast to other studies [14,15], our data show *A. baumannii *is an important pathogen isolated in respiratory samples of B-NP patients and is also an independent risk factor for bacteremia. *A. baumannii *has a high level of antibiotic resistance, but with a low virulence [25,26]. A recent study that compared risk factors and outcomes for bacteremia due to *A. baumannii *and *Klebsiella pneumoniae *showed bacteremia due to *A. baumannii *was significantly more frequent secondary to NP than bacteremia due to *K. pneumoniae *[27]. Jamulitrat and colleagues showed that the observed higher mortality rate among patients with an imipenem-resistant *A. baumannii *bloodstream infection might not be attributable to imipenem resistance but in some part may be due to a more severe illness, inappropriate antimicrobial therapy, and primary source of infection [28].

There are several factors linked with MRSA isolation in VAP episodes: administration of antibiotics before the development of VAP [29,30] and the length of hospital stay rather than the period of mechanical ventilation were strongly associated with MRSA isolation [31]. Methicillin resistance represents an independent risk factor for a poor outcome, prolonged hospitalization and high hospital costs in VAP episodes [32], even when therapy was appropriate [33]. Interestingly, new antimicrobial development and novel anti-adherence tools based upon fibrinogen-binding protein derivatives [34] might provide new opportunities to improve survival by preventing bacteremic nosocomial pneumonia [35]. The time to initiation of appropriate therapy with a molecular analysis of MRSA isolates and virulence factors would be useful in future research.

Our results show that there is an independent association between MRSA and *A. baumannii *etiology and development of bacteremia in NP patients; but mortality is associated with bacteremia and severity of disease. These results confirm the concept shown in the study by Agbaht and colleagues, since they also found an independent association between MRSA and bacteremia but mortality was associated with bacteremia rather with MRSA [6]. In addition, the presence of bacteremia has been identified as an independent risk factor for mortality by other authors and included in clinical scores for severity assessment of VAP episodes [36].

The present study has several strengths. Data were generated from a multi-institutional study and represent an interesting sampling from different European ICUs. Our study enrolled patients prospectively and represents a homogeneous population from critical care and mechanically ventilated patients. The original approach from our study was to consider all HAP episodes for analysis since patients, especially those with VAP, have a high chance of multiple drug-resistant pathogens, prior antibiotic therapy and multiple co-morbidities.

The present study also has several potential limitations that should be addressed. This study was observational and non-interventional, in which the participating 27 ICUs from 9 countries were self-selected. The prescription of antibiotics was chosen in accordance with the protocol agreed by the institution. Second, the decision to extract blood cultures was chosen in accordance with local protocols and the physician's clinical decision. Although not all patients who developed NP underwent blood cultures, in the present analysis two out of three patients' blood cultures were subsequently obtained. There was case-mix difference between the participating centers, but all types of ICU were represented with no statistical differences found among bacteremic episodes. We acknowledge that a matched cohort would be more powerful to identify independent risk factors associated with bacteremic episodes. Our analysis included, however, in a backward logistic regression model, all variables identified in univariate analysis and adjusted for severity of disease. Although potential unknown confounding factors might be present, our model presented an adequate goodness of fit.

## Conclusions

The present study suggests that predisposition factors such as diagnostic category at admission and infection-related factors such as etiology are associated with higher risk for B-NP. Recognition of these risk factors is relevant for clinical practice, as bacteremia is an independent risk factor for worse outcome in intubated patients with NP. Our findings support the need to perform blood cultures in hospitalized patients with NP.

## Key messages

• A total 14.6% of episodes of NP in the European ICUs are bacteremic.

• The main pathogens isolated in blood cultures of B-NP patients are MRSA and *A. baumannii*.

• Bacteremia is independently associated with a higher mortality rates in patients with NP.

• B-NP episodes are more frequent in patients with medical admission, MRSA and *A. baumannii *etiology, and prolonged mechanical ventilation.

## Abbreviations

B-NP: bacteremic nosocomial pneumonia; CI: confidence interval; HAP: hospital-acquired pneumonia; ICU: intensive care unit; MRSA: methicillin-resistant *Staphylococcus aureus*; NB-NP: nonbacteremic nosocomial pneumonia; NP: nosocomial pneumonia; OR: odds ratio; SAPS: Simplified Acute Physiology Score; VAP: ventilator associated pneumonia.

## Competing interests

The authors declare that they have no competing interests.

## Authors' contributions

MM made substantial contributions to the intellectual content of the paper in acquisition, analysis and interpretation of data, drafting of the manuscript, critical review of the manuscript for important intellectual content and statistical analysis. TL contributed with conception and design, analysis and interpretation of data, drafting of the manuscript, critical review of the manuscript for important intellectual content and statistical analysis. IM-L contributed to acquisition, analysis and interpretation of data, drafting the manuscript and critical review of the manuscript for important intellectual content. RM, MN, HW, SC, ED and DK contributed to acquisition of data and critical review of the manuscript for important intellectual content. JR contributed to the conception and design, critical review of the manuscript for important intellectual content and supervision. All authors read and approved the final manuscript.
